# Adult Limbal Neurosphere Cells: A Potential Autologous Cell Resource for Retinal Cell Generation

**DOI:** 10.1371/journal.pone.0108418

**Published:** 2014-10-01

**Authors:** Xiaoli Chen, Heather Thomson, Jessica Cooke, Jennifer Scott, Parwez Hossain, Andrew Lotery

**Affiliations:** 1 Clinical and Experimental Sciences, Faculty of Medicine, University of Southampton, Southampton General Hospital, Southampton, United Kingdom; 2 Eye Unit, University Hospital Southampton NHS Foundation Trust, Southampton, United Kingdom; 3 School of Clinical Sciences, University of Bristol, Bristol Eye Hospital, Bristol, United Kingdom; Queen’s University Belfast, United Kingdom

## Abstract

The Corneal limbus is a readily accessible region at the front of the eye, separating the cornea and sclera. Neural colonies (neurospheres) can be generated from adult corneal limbus *in* *vitro*. We have previously shown that these neurospheres originate from neural crest stem/progenitor cells and that they can differentiate into functional neurons *in* *vitro*. The aim of this study was to investigate whether mouse and human limbal neurosphere cells (LNS) could differentiate towards a retinal lineage both *in* *vivo* and *in* *vitro* following exposure to a developing retinal microenvironment. In this article we show that LNS can be generated from adult mice and aged humans (up to 97 years) using a serum free culture assay. Following culture with developing mouse retinal cells, we detected retinal progenitor cell markers, mature retinal/neuronal markers and sensory cilia in the majority of mouse LNS experiments. After transplantation into the sub-retinal space of neonatal mice, mouse LNS cells expressed photoreceptor specific markers, but no incorporation into host retinal tissue was seen. Human LNS cells also expressed retinal progenitor markers at the transcription level but mature retinal markers were not observed *in* *vitro* or *in* *vivo*. This data highlights that mouse corneal limbal stromal progenitor cells can transdifferentiate towards a retinal lineage. Complete differentiation is likely to require more comprehensive regulation; however, the accessibility and plasticity of LNS makes them an attractive cell resource for future study and ultimately therapeutic application.

## Introduction

Retinal diseases are the leading cause of untreatable blindness worldwide. These conditions include age related macular degeneration (AMD) and a wide spectrum of inherited retinal diseases. Irreversible visual impairment arises due to a gradual loss of light sensory neurons- photoreceptors and/or their supportive cells the retinal pigment epithelium (RPE). Unlike lower vertebrates, adult mammals cannot regenerate retinal neurons. The visual disability caused by these diseases carries a formidable clinical and socio-economic burden in western countries [1].

Cell based therapies are an attractive approach to treat retinal disease [2]. They offer the potential to restore functional vision. Recent studies have demonstrated that transplanted photoreceptor precursor cells can form synaptic connections with host retina and improve visual function in animal models of retinal degeneration [2]–[4]. However, identifying practical cell sources to generate sufficient functional cells for transplantation remains challenging. Utilizing embryonic or fetal tissue is difficult due to limited resources, ethical issues or risks of tumour formation [5]. In addition, transplant rejection may occur due to chronic immune responses. This has been observed after transplantation with a 90% loss of integrated allogeneic photoreceptors by 4 months, and nearly 100% loss at 6 months [6]. Therefore immune-matched autologous cell resources have considerable advantages.

Autologous somatic cells can be genetically reprogrammed into induced pluripotent stem cells (iPSCs), an embryonic stem cell-like state, and then differentiate into all three germ layer cells, including a retinal lineage with the production of photoreceptors and RPE cells [7]. These iPSCs derived cells have been transplanted into animal models of retinal degeneration and have shown promising results [8], [9]. Whilst using this differentiation method, risk of tumour formation remains due to contamination with undifferentiated cells [10]. Recently, a new 3D culture method has successfully produced a larger number of “integration-competent” photoreceptor cells from ESCs. The process of differentiation recapitulates the *in* *vivo* development of the optic-cup [11], [12]. This 3D culture protocol is also based on Matrigel, a solubilised basement membrane derived from murine sarcomas. It contains undefined xenogenic growth factors, which prevents the protocol from production of clinical grade transplantable retinal cells. Hence, potential adverse effects still need to be carefully addressed prior to iPSCs based cell therapy.

Adult stem/progenitor cells are an attractive alternative autologous cell resource. Studies have shown the plasticity of these cell types. They can be induced to transdifferentiate toward lineages other than that of their origin [13]–[15]. Certain cell types can also de-differentiate into multipotent progenitor cells that give rise to cells that express retinal specific markers. This includes ciliary body (CB) epithelium and retinal Müller glial (MG) cells, although their potential remains controversial [16]–[21]. In addition, routine safe and practical surgical techniques do not exist to harvest them. Therefore they are unlikely to be a practical autologous cell resource in the immediate future.

In contrast, the corneal limbus is a readily accessible area, where the superficial layers are amenable to tissue harvesting. Several groups have reported generation of neural colonies (neurospheres) from cornea/limbus by neurosphere assay [22], [23]. This utilises a well-defined suspension culture system, thus it is more appropriate for the derivation of cells for clinical application. Zhao *et al*. showed the rat limbal cell cultured as neurospheres expressed photoreceptor specific markers following co-culture with neonatal retinal cells. The co-culture condition provides a photoreceptor promoting microenvironment [15]. However, it remains unknown whether LNS from other species, particularly from humans and mice, can give rise to retinal like cells. Their ability to generate photoreceptor like cells *in vivo* and to integrate into host retina is yet to be proven. In addition, the number of adult stem/progenitor cells normally decreases with age. It is thus important to investigate whether LNS can be cultured from aged human eyes and used as an autologous cell resource in age related diseases. Here, we investigate LNS derived from mice and humans to extend the knowledge of limbal cells to other species.

We have previously conducted a comprehensive characterization of mouse LNS regarding their self-renewal capacity, origin and ultrastructure, and shown that neurospheres derived from the corneal limbus are neural crest derived limbal stromal stem/progenitor cells. For the first time, we demonstrated that functional neural-like cells can be derived from neural crest-derived limbal cells [24]. The aim of this study is now to investigate whether mouse and human limbal neurosphere cells (LNS) can differentiate into retinal like cells both *in* *vivo* and *in* *vitro* after exposure to a developing retinal microenvironment.

## Materials and Methods

### Animals

The use of animals in this study was in accordance with the ARVO statement for the use of animals in Ophthalmic and Vision Research and the regulations set down by the UK Animals (Scientific Procedures) Act 1986. The protocol was approved by the UK Home Office. All surgery was performed under isoflurane inhalation anaesthesia, and every effort was made to minimize suffering.

Male C57BL/6 mice were maintained in the animal facility of the University of Southampton. Adult mice (6–8 weeks old) were used for corneal limbal cell culture, differentiation, and transplantation studies. Postnatal (PN) day 1–3 mice were used for isolation of retina to provide a conditioned retinal development environment *in* *vitro* and as recipients for sub-retinal transplantation of LNS cells.

### Cell culture

Human limbal tissues that were consented for research use were requested from the Corneal Transplant Service Eye Bank in Bristol (CTS Eye Bank, http://www.bristol.ac.uk/clinical-sciences/research/ophthalmology/tissue-bank/eye-bank/). The study was approved by Southampton & South West Hampshire Research Ethics Committee (A). The use of human fetal retinas followed the guidelines of the Polkinghome Report, and was approved by the Southampton & South West Hampshire Local Research Ethics Committee. Written informed consent from the donor or the next of kin was obtained for use of human samples in this research.

Adult mouse/human corneal limbal cells were cultured as previously described [15], [23], [24]. In brief, mouse limbal tissue was digested with 0.025% (w/v) trypsin/EDTA (Sigma-Aldrich, Ayrshire, UK) at 37°C for 10–12 min, and then in 78 U/ml of collagenase (Sigma-Aldrich) and 38 U/ml of hyaluronidase (Sigma-Aldrich) for 30 min. Human limbal tissue (age 72–97 years) was incubated in collagenase (78 U/ml) and hyaluronidase (38 U/ml) in M2 medium (Sigma-Aldrich) at 37°C overnight. Dissociated cells were cultured at a density of 1×10^5^ in DMEM: F12GlutaMAX (Invitrogen, Dorset, UK) supplemented with 2% B27 (Invitrogen), 20 ng/ml of EGF (Sigma-Aldrich) and 20 ng/ml of FGF2 (Sigma-Aldrich).

To promote neurosphere cell differentiation towards photoreceptors, co-culture was conducted as previously described [24]. In brief, sphere cells derived from adult corneal limbus were plated onto Poly-D-Lysine (P-D-L) (Sigma-Aldrich) and laminin (Sigma-Aldrich) coated wells and co-cultured with dissociated PN1-3 mouse retinal cells or fetal week (Fwk) 7–8 human fetal retinal cells using Millicel CM inserts (pore size 0.4 µm; Millipore, Watford, UK) for 1–2 weeks. A papain dissociation system was used to dissociate retinal cells as per manufactures’ instructions (Worthington-Biochemical, Berkshire, UK). In brief, minced retinal tissue was incubated with a mixture of papain (20 units/ml) and DNase (0.005%) for approximately 40 min at 37°C. Enzymatic digestion was stopped using inhibitor solution contained 10% ovomucoid and 0.005% DNase. Cell suspensions were then filtered using 100 µm cell strainers (BD Falcon, Oxford, UK) to eliminate large cell clumps. Cell pellets were resuspended into neural differentiation medium for co-culture. Neural differentiation medium was Neurobasal A media (Invitrogen), 2% B27, 0.5 mM L-Glutamine (Sigma-Aldrich), 0.5–1% fetal bovine serum (FBS, Sigma-Aldrich), 1 µM retinoic acid (RA, Sigma-Aldrich) and 1 ng/ml brain-derived neurotrophic factor (BDNF, R&D System, Abingdon, UK). In human LNS differentiation condition 2, Sonic hedgehog (Shh, 3 nM, R&D Systems), Taurine (1 mM, Sigma-Aldrich) and RA (1 µM) were added into Neurobasal A media containing 2% B27, 0.5 mM L-Glutamine and 0.5–1% FBS. Half of the medium was changed every other day.

### Immunocytochemistry/immunohistochemistry

Cells were fixed with 4% Paraformaldehyde (PFA, pH 7.4, Sigma-Aldrich) for 15–20 min at 4°C. Cells or tissue slides were permeabilized and blocked with 0.1 mM phosphate buffer saline (PBS) supplemented with 0.1% Triton X-100 (Sigma-Aldrich) and 5% donkey blocking serum (DBS, Sigma-Aldrich) for 0.5–1 hrs at room temperature (rt), prior to addition of primary antibodies. Specific IgG secondary antibodies (Alexa Fluor 488, 555-conjugate (1∶500) Invitrogen) were incubated at rt for 1–2 hrs. Negative controls omitted the primary antibody. Nuclei were counterstained with 10 ng/ml 4′, 6′-diamidino-2-phenylindole (DAPI, Sigma-Aldrich). Images were captured using a Leica DM IRB microscope or a Leica SP5 confocal laser scanning microscope (Leica Microsystems UK Ltd, Buckinghamshire, UK). To quantify the percentage of cells expressing a particular phenotypic marker, the number of positive cells was determined relative to the total number of cells (DAPI labelled nuclei). A total of 500–1000 cells from 6 random fields were counted per marker. The antibodies used are listed in Table S1 in File S1.

### Transmission electron microscopy (TEM)

Samples were fixed with 0.1 M sodium cacodylate (Sigma-Aldrich), 3% glutaraldehyde (Sigma-Aldrich), 4% PFA and 0.1 M PIPES buffer (Sigma-Aldrich) for 15 min. After rinsing in 0.1 M PIPES buffer, samples were postfixed in 1% buffered osmium tetroxide (1 hr, Sigma-Aldrich) and block stained in 2% aqueous uranyl acetate (20 min, Sigma-Aldrich). Following dehydration through a graded series of ethanol fixations up to 100%, the samples were embedded in TAAB resin (TAAB Laboratories, Berkshire, UK). Gold sections were cut on a Leica OMU 3 ultramicrotome (Leica), stained with Reynolds lead stain and viewed on a Hitachi H7000 transmission electron microscope equipped with a SIS megaview III digital camera (Hitachi High-Technologies Corporation, Berkshire, UK).

### Reverse transcription-polymerase chain reaction (RT-PCR)

Total RNA was isolated and cDNA synthesis was performed as per manufacturer’s instructions using an RNeasy Plus kit (Qiagen, West Sussex, UK) and High Capacity cDNA Reverse Transcription Kits (Applied Biosystems, Cheshire, UK). cDNA was amplified using gene specific primers (Table S2 in File S1) using step cycles (denaturing for 30 sec at 94°C; annealing for 30 sec at 60°C and extension for 30 sec at 72°C for 35 cycles unless indicated otherwise). Electrophoresis was performed on a 1.5% agarose gel. Real time quantitative PCR experiments were performed using Rotor-gene 6000 (Qiagen, Manchester, UK). Primers and FAM-labeled probes were designed and manufactured by PrimerDesign (PrimerDesign Ltd, Southampton, UK; Table S3 in File S1). Other PCR reagents and amplification protocol were obtained from the same commercial provider. Samples were analysed in duplicate and normalised to Gapdh expression level by the 2^−ΔΔCt^ method.

### Cell transplantation

LNS cells passages 3–5 were used for transplantation. Following transfection with a lentiviral-eGFP vector (kind gift from Professor Andrew Dick, University of Bristol) at a concentration of 5 MOI (multiplicity of infection), Green fluorescent protein (GFP) was observed in over 95% of LNS cells 72 hours post-transfection. LNS cells were dissociated into single cells with Accutase (Sigma-Aldrich), washed twice with PBS and resuspended at a concentration of 4,000–10,000 cells/µl in DMEM media (Invitrogen). P1–3 mice were subjected to inhalation anaesthesia using 50% isoflurane (Sigma-Aldrich) mixed with 50% oxygen. Animals received cell transplants (0.8–1.0 µl) via a transcleral injection into the subretinal space using a 34 gauge hypodermic needle (Hamilton, Switzerland), connected to a Hamilton syringe (Hamilton). Needle insertions were made tangentially though the lateral superior sclera, and cells were injected slowly to produce retinal detachments. Mice were sacrificed 2–3 weeks after transplantation. After enucleation, the eyes were fixed with 4% PFA in PBS, and cryoprotected in 20% sucrose, before embedding in OCT (TissueTek, Thatcham, UK). Cryosections (16 µm thick) were cut and affixed to poly-L-lysine coated slides (Thermo Scientific, Hertfordshire, UK).

### Statistical Methods

All results are presented as mean ± SEM (standard error of the mean) unless otherwise stated; *n* represents the number of replicates. Statistical comparisons were made using a Student’s t-test or one way analysis of variance (ANOVA) with a significance threshold of p<0.05. GraphPad Prism Software (GraphPad, San Diego, USA) was used for statistical analysis.

## Results

### Generation of neurospheres from adult mouse and human limbal cells

We previously demonstrated that neural colonies (neurospheres) can be generated from mouse adult corneal limbus in serum free medium in the presence of mitogens [24].

**Figure 1:**
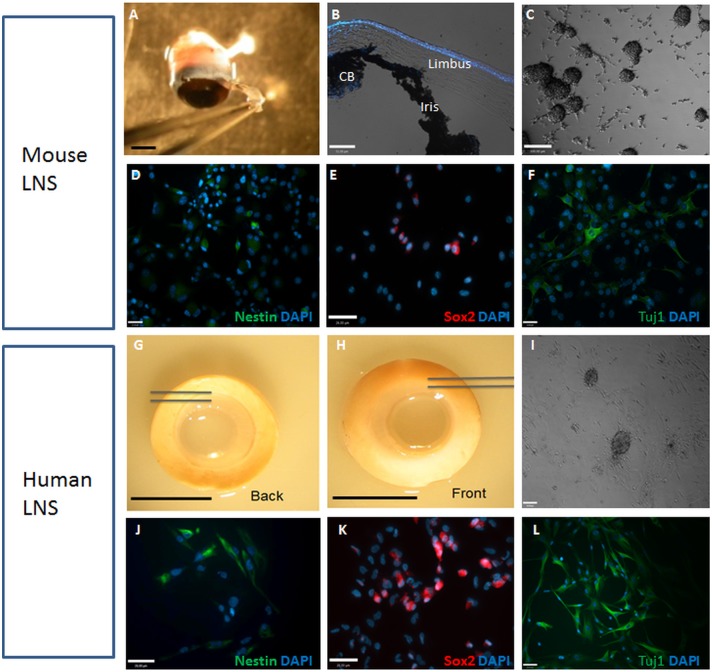
Generation of neurospheres from adult mouse and human limbal cells. Upper panel: (A–B) illustrates the murine limbal region used for generation of LNS. (C) Phase contrast image of adult mouse LNS. (D–F) LNS cells expressed neural stem/progenitor cell markers nestin, Sox2 and Tuj1. Lower panel: Human donor limbal rim (from age 72–97 years) were obtained after corneal graft surgery (G–H). Limbal region between the two lines was used for generation of LNS. (I) Phase contrast image of human LNS. (J–L) LNS cells expressed neural stem/progenitor cells marker nestin, Sox2 and Tuj1. Scale bar: 1 mm (A), 50 µm (B, I), 100 µm (C), 26 µm (D–F & J–L) and 10 mm (G–H).

By using the same culture system, we sought to enrich the neural stem-like cells from both adult mouse and aged human corneal limbus. Limbal tissue was harvested from adult mice (6–8 weeks, Fig. S1 in File S1, File S2) and aged donors (72–97 years of age), and cultured in the serum free neurosphere culture system. The mouse and human limbal sphere-clusters started forming on day 5 and day 7 *in* *vitro,* respectively. Approximately 100–120 LNS, size ranging from 50–150 µm in diameter, were generated from aged individual human eyes after 10–14 days (Fig. 1). The numbers were significantly less than those generated from single young adult mouse eyes (392±18, p<0.001). This may be due to the age of the human donor eyes as well as low cell viability after 5–28 days tissue storage at the local eye bank. We examined the phenotype of the cells within the human LNS. As revealed by immunocytochemistry, human limbal sphere clusters expressed neural stem cell markers, including the transcription factor Sox2 and intermediate filament protein nestin. The proportions of Sox2 and nestin positive cells were 31.2±10.2% and 34.8±2.2% respectively (Fig. 1). This is similar to our previous findings in mouse LNS [24]. Co-expression of both markers was observed in 26.3±9.7% of cells.

### Expression of photoreceptor specific markers in mouse LNS cells following co-culture with developing retinal cells

**Figure 2:**
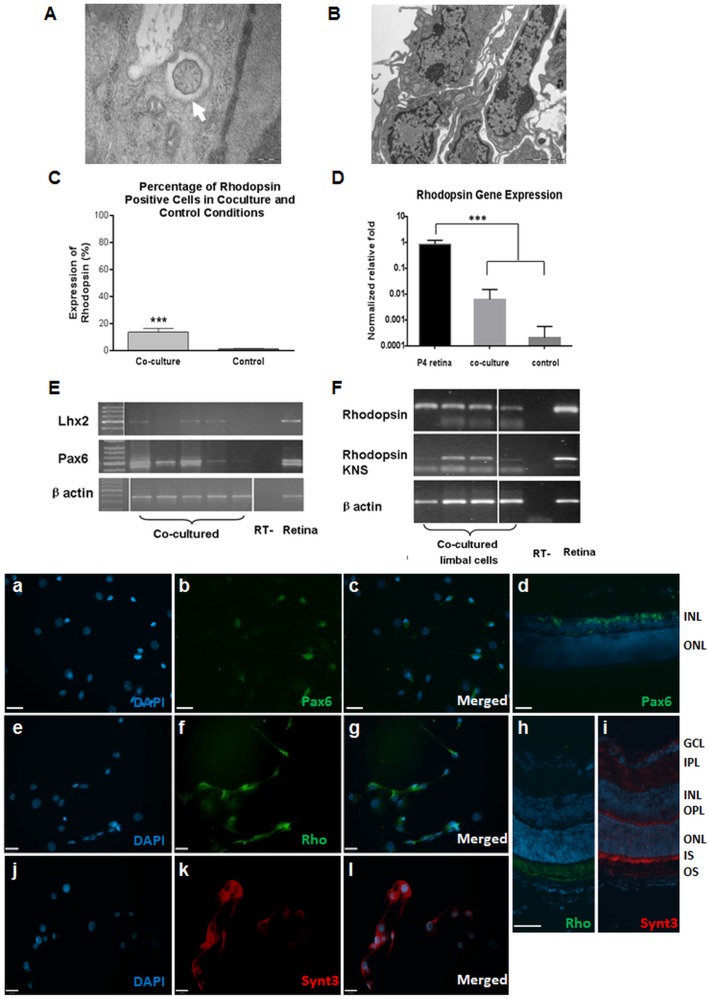
Mouse LNS cells differentiated towards retinal lineage following co-culture with developing retinal cells *in* *vitro*. TEM of mouse LNS cells following differentiation (A–B): 9+0 non-motile cilia (A, arrow), were present in induced limbal cells, whilst cell junctions were not apparent (B). Histograms illustrating the percentage of rhodopsin positive cells (C, Mean ± SEM, t test, P<0.001) and RT-qPCR on gene expression level (D) in mouse LNS cells following differentiation in co-culture vs control condition (Mean ± SEM, ANOVA, P<0.001). Retinal progenitor cell markers & photoreceptor specific genes were detected by RT-PCR (E–F) and immunocytochemistry (a–c, e–g, j–l). Mouse retinal sections were used as positive controls for antibody specificity tests (d, h, i); Cell nuclei were counter stained in blue with DAPI. Rho: rhodopsin, Synt3: Syntaxin3, GL: ganglion layer, OPL: Outer plexiform layer, INL: inner nuclear layer, OPL: Outer plexiform layer, ONL: outer nuclear layer, OS: outer segments, IS: inner segments Scale bar: 200 nm (A–B); 13 µm (a–c, e–g, j–l); 26 µm (d, h, i).

To promote differentiation of LNS into photoreceptor like cells, mouse LNS cells were co-cultured with neonatal retinal cells. As previously described, cultured PN1–3 murine retinal cells release diffusible rod promoting factors, which can promote stem cell differentiation or transdifferentiation towards rod photoreceptors [25], [26]. Cell inserts with a semi-permeable membrane were utilised to avoid cell contamination. Mouse LNS cells formed a monolayer following withdrawal of mitogens, with cells displaying neural morphologies. Expression of retinal progenitor cell markers Pax6, Lhx2 was observed by RT-PCR after 2–4 days of co-culture. Following 7–10 days in co-culture, mouse LNS cells were immunopositive for the photoreceptor specific marker Rhodopsin (13±3%) (Fig. 2). Rhodopsin positive cells had a dendritic morphology, with staining in both the cell body and cytoplasmic processes. Under control conditions (without co-culture), a few Rhodopsin positive cells were detected (1.19±0.39%). Statistical analysis showed a significant difference between the two groups in the presence and absence of co-culture (P<0.001, unpaired t-test). Expression of both Rhodopsin and Rhodopsin kinase was also detected at the RNA level by RT-PCR (Fig. 2), although the expression level was significant lower than native neonatal retinal tissues (∼1%, P<0.001, ANOVA). Approximately 8–10% of cells exhibited strong immunoreactivity to Syntaxin3, a major component of synapses within the retina (Fig. 2) [27].

Differentiated LNS cells displayed ultrastructural changes following co-culture. We previously reported that cellular junctions such as gap junctions and immature adherens junctions were found in LNS [24]. TEM revealed loss of junctions within the LNS and presence of non-motile primary cilia (Fig. 2A, arrow) after differentiation. The cilia noted in co-cultured LNS were identified as sensory cilia, consisting of an axoneme of nine doublet microtubules, with a lack of a key central pair of microtubules that are involved in ciliary motility [28]. Although not specific to retinal lineage cells, this subtype of sensory cilia is present in photoreceptor and RPE cells.

### Potential of mouse LNS cells differentiation to retinal lineage *in* *vivo*

To investigate the differentiation of mouse LNS cells *in* *vivo*, eGFP expressing donor cells between passage 3–5 were transplanted into the sub-retinal space (SRS) of P1 wild-type C57BL/6 mice. P1 mice were selected as hosts since their retinas were undergoing rod photoreceptor genesis. This produces an optimal microenvironment to stimulate cell differentiation and integration [25].

**Figure 3:**
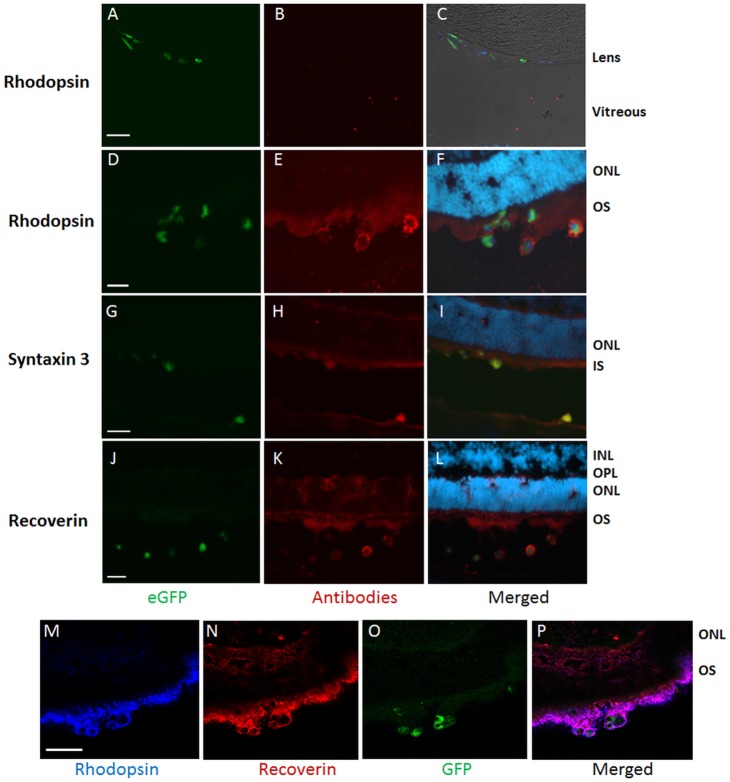
Expression of photoreceptor specific markers in mouse LNS cells following transplantation into the sub-retinal space (SRS) of P1–3 mice. Immunohistochemistry was conducted on mouse eyes 2 weeks after injection of mouse LNS. eGFP tagged LNS cells were present in the vitreous or SRS (green, A, D, G, J); Mouse LNS cells located in the vitreous cavity, incorporated into the lens epithelium, but did not express rhodopsin (B, C). Photoreceptor specific markers were observed in donor cells (green) and host retinal tissue (red, E, H, K). Cell nuclei were counter stained with DAPI (blue) in the merged images (C, F, I, L). Three-channel merged images showed co-expression of rhodopsin and recoverin in donor cells and host retina (M-P). OPL: Outer plexiform layer, ONL: outer nuclear layer, INL: inner nuclear layer, OS: outer segments, IS: inner segments. Scale bar: 26 µm (A, D, G, J); 50 µm (M).

Following injection of a suspension of dissociated LNS cells, identified by their eGFP tags, grafted mouse LNS cells were found in the SRS or the vitreous (Fig. 3). Donor cells did not appear to migrate into the host retina. Mouse LNS cells located in the SRS showed small round cell bodies. Immunohistochemical analysis using photoreceptor specific antibodies against rhodopsin, recoverin and syntaxin3 demonstrated expression of these markers in eGFP cells, indicating differentiation of mouse LNS cells along a photoreceptor lineage *in* *vivo*. This concurs with our observations following *in* *vitro* co-culture using mouse LNS cells. Interestingly, mouse LNS cells located in the vitreous cavity, incorporated into the lens epithelium. However, photoreceptor markers were not detected in these eGFP tagged LNS cells (Fig. 3). This was possibly due to the “non-permissive” environment of the vitreous cavity.

### Potential of human LNS cell differentiation towards retinal lineages *in* *vitro* and *in* *vivo*

**Figure 4:**
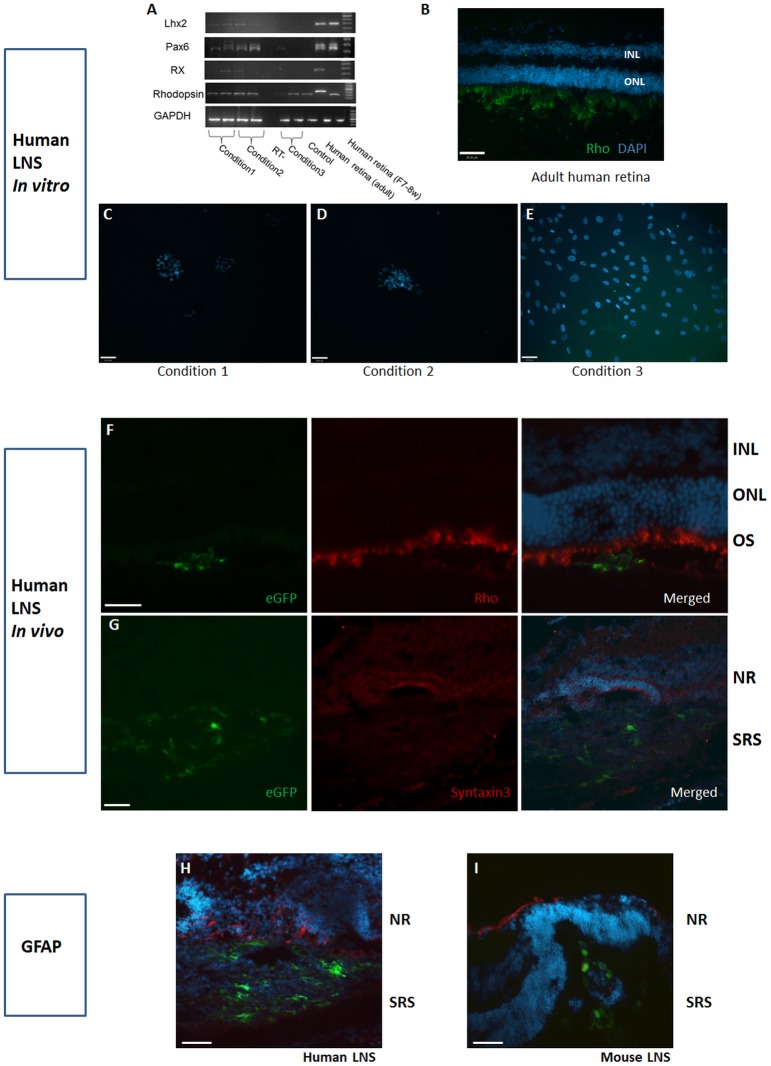
Mature retinal lineage markers were not detected in human LNS derived cells *in* *vitro* or *in* *vivo*. Upper panel: RT-PCR and immunocytochemistry were performed on human LNS cells following culture under retinal differentiation promoting conditions. Condition 1: human LNS cells co-cultured with mouse developing retinal cells; Condition 2: human LNS cells cultured in differentiation media in the presence of Shh/RA/Taurine. Condition 3: human LNS cells co-cultured with human fetal retina (post conception 56 days). (A) Retinal progenitor markers Pax6, Lhx2, Rx were detected in 50% of samples, but the mature photoreceptor marker rhodopsin was absent in all conditions and also in fetal retina. Human adult retinal cDNA was used as a positive control for photoreceptor specific genes. Negative control (RT-) omitted reverse transcriptase. (B) Retinal sections were used as positive controls for immunostaining. (C–E) Rhodopsin was not detected in human LNS cells when co-cultured with mouse/human developing retinal cells, or when culture conditions were supplemented with extrinsic factors Shh/Taurine/RA. (F–H): human LNS cells were transfected with LVV-eGFP (green) and transplanted into the sub-retinal space (SRS) of neonatal mice. Rhodopsin and Syntaxin3 were not detected in cells grafted into the SRS. Upregulation of GFAP was observed in host retina at the graft sites. (I) GFAP upregulation was less apparent in host retina receiving allogeneic mouse LNS cells (green). Cell nuclei were counter stained with DAPI (blue) in the merged images. Shh: Sonic hedgehog, RA: Retinoic acid. Rho: Rhodopsin, Sytx3: Syntaxin3, GFAP: Glial fibrillary acidic protein, ONL: inner nuclear layer, ONL: outer nuclear layer, RPE: retinal pigment epithelium, NR: neural retina; scale bar: 50 µm (B), 26 µm (C–I).

To investigate the potential of human LNS cells to transdifferentiate towards retinal-like cells, both neonatal mouse retinal cells and early human developing retinal cells were used for the co-culture assay. In addition, the previously reported extrinsic factors Shh/Taurine/RA were utilised to promote cell differentiation to retinal cells [15], [29]. Low levels of Lhx2 and Pax6 were detected in all samples co-cultured with P1 mouse retinal cells or Shh/Taurine/RA conditions. The retinal homeobox gene (Rx) was expressed in 50% of the above samples. On the contrary, human LNS cells co-cultured with early developing human retinal cells or in control conditions where only differentiation media was applied, did not express Lhx2 or Pax6 (Fig. 4A, C–E). Mature photoreceptor specific markers such as Rhodopsin were not detected in human LNS cells at either the transcript or protein level as shown by RT-PCR and immunocytochemical analysis.

We further investigated whether the *in* *vivo* permissive environment of neonatal mouse retina could induce human LNS cells to differentiate into retinal like cells. Following transduction of cells with eGFP, human LNS were subsequently transplanted into the SRS of wildtype PN1-3 mice. The human xenografts survived for 25 days in the absence of immuno-suppression (longest observation time). They formed cell clusters, and displayed dendritic processes. However, their processes didn’t extend towards the ONL, but mainly spread horizontally. Adjacent to the site where human LNS cells were grafted, host retinas displayed an upregulation of glial fibrillary acidic protein (GFAP) and an aberrant host retinal structure. This was more severe than in the recipient retinas transplanted with allogeneic mouse LNS cells. The histology suggested formation of a glial scar due to an immune response to the xenograft. Similarly with co-cultured human LNS cells, photoreceptor markers, such as rhodopsin and recoverin, were not detected in any grafted human eGFP-LNS cells (10 eyes, Fig. 4). This suggests that human LNS cells failed to differentiate towards a photoreceptor lineage following transplantation into the SRS of developing mouse eyes. This implies that more comprehensive intrinsic and extrinsic regulation is required to drive human LNS cells differentiation towards retinal-like cells.

## Discussion

We have previously demonstrated that adult mouse LNS are neural crest-derived limbal stromal stem/progenitor cells [24]. They can generate functional neural-like cells *in* *vitro* [24]. We now report that these cells have the potential for differentiation towards a retinal lineage. Following co-culture with neonatal retinal cells, LNS cells express retinal progenitor cell markers such as Lhx2 and Pax6, and mature retinal specific markers including Rhodopsin and Rhodopsin Kinase, with approximately 10% of cells becoming immunopositive for rhodopsin. The expression of photoreceptor specific markers at a transcript level indicates endogenous expression of these photoreceptor specific genes. Moreover, the major retinal synapse component syntaxin3 and sensory cilia were also observed, as would be expected in differentiated retinal cells. After transplantation into the SRS, expression of rhodopsin, recoverin and syntaxin3 were also detected in grafted LNS cells. For the first time, this work demonstrates that neural crest-originated limbal stromal stem/progenitor cells have the potential to generate retinal-like cells both *in* *vitro* and *in* *vivo*.

We further investigated whether LNS can be generated from aged human eyes, and whether they have a similar potential to generate retinal cells. We generated LNS from aged human limbal tissue from donors up to 97 years of age. The upregulation of retinal progenitor markers such as Lhx2, Pax6 and Rx was noted following culture in permissive conditions *in* *vitro*. To the best of our knowledge, this is the first evidence showing that human LNS have the plasticity to express retinal progenitor markers. However, mature photoreceptor markers were not observed. The lack of further cell maturation suggests that more comprehensive intrinsic and extrinsic regulation is needed cf. mouse cells. Extrinsic factors released by PN1–3 mouse retinal cells may be insufficient in promoting further differentiation of human cells towards a mature retinal cell lineage.

We also co-cultured human LNS with fetal week (Fwk) 7–8 human retinal cells. LNS did not appear to differentiate towards a retinal lineage in this circumstance. This may be due to the fact that retinal tissue/cells at the gestational stage of Fwk 7–8, have not started rod genesis [30]. Our observation is consistent with a previous report that rod promoting activity is only observed in retinal cells at the peak of rod genesis, but not at an early developmental stage or in adult retinal cells [25]. Due to ethical concerns, we were unable to access later stage human fetal retinal tissues. However, it is encouraging that human LNS expressed retinal progenitor markers when exposed to several defined factors including Shh, Taurine and RA. Shh has been shown to be involved in the formation of the ventral optic cup, specification of dorso-ventral polarity in the optic vesicle, and governing of ocular morphogenesis [31]. Besides specification of the eye field during embryonic development, Shh also has been implicated in the control of retinal development in vertebrates [32], [33] and is required for the maintenance of retinal progenitor cell proliferation [34]. Another factor, RA, plays an important role in early eye development as well as in the differentiation, maturation and survival of photoreceptors [35]. Similar to the effect of co-culture with neonatal (P1–3) mouse retinal cells, the combination of Shh, Taurine and RA promoted upregulation of retinal progenitor markers in human LNS. This suggests that defined culture conditions may replace the use of animal tissue in the future.

We did not observe LNS cell migration or integration into the host retina following sub-retinal transplantation into neonatal mice. Cell integration into the retina remains challenging. Despite being derived from the same origin as neural retina, iris or CB derived cells have also shown limited ability for retinal integration [36]. The proportions of cells which integrate into embryonic retinal explants or retina from degenerate animal models are small. Studies using retinal progenitor cells from embryonic retina have also shown little integration into host retina, although mature retinal phenotypes have been observed following sub-retinal transplantation [37], [38]. MacLaren *et al.* investigated the optimal cell resource for functional integration into adult retina [2], [6]. The cells which migrated and integrated were shown to be post-mitotic rod precursor cells. Therefore, the ontogenetic stage of transplanted cells is important for successful cell integration. Grafted LNS cells in this study were not fully committed post-mitotic cells. This may explain why cell integration was not observed. The host microenvironment is also essential for inducing cell differentiation and migration. In a study involving transplantation of IPE derived cells [39], the grafted cells expressed the photoreceptor specific marker rhodopsin when they were transplanted into the SRS of embryonic chicken (E5) eyes. On the contrary, they did not express rhodopsin or other neural markers when they were transplanted into the vitreous cavity. This is in accordance with our observation that the LNS cells transplanted into the vitreous do not express photoreceptor markers.

LNS display plasticity, the potential to cross the tissue/germ layer boundary and generate cells other than their origin [15], [24]. However, LNS have limited potential to generate photoreceptor-like cells. The highest rhodopsin expression level noted using LNS derived cells was ∼3% of that observed compared to using neonatal mouse retinal tissue. Reports on other ocular stem-like/progenitor cells also show limited success in the generation of photoreceptor regardless of cell origin. Recently two independent groups showed CE-derived cells failed to give rise to photoreceptor cells [16], [17]. Retinal neurosphere cells derived from neonatal mice also had a low efficiency (1–2%) in generation of rhodopsin positive cells during spontaneous differentiation [40].

It has been suggested that cell reprogramming is likely to be needed for robust photoreceptor cell production. LNS cells would also be an optimal cell resource for reprogramming and/or trans-differentiation and subsequent retinal repair. They are readily accessible, highly proliferative and multipotent ocular stem cells. iPSCs have been generated from mouse and human somatic cells by ectopic expression of four transcription factors including OCT4, SOX2, c-Myc and KLF4. Due to risks such as insertional mutagenesis or tumour formation, it is desirable to use the minimal number of transcription factors and to eliminate oncogenic factors [41]–[43]. This goal can be achieved through optimal selection of candidate cell resources. For example, Kim *et al*. generated iPSCs from adult mouse and human neural stem cells by ectopic expression of a single transcription factor Oct4 [41]–[43]. As we previously demonstrated [24], a diverse range of neural stem markers including Sox2, were detected on LNS cells. The multipotent capability of limbal stroma derived stem/progenitor cells have been reported by different research groups [22], [23], [44]–[46]. Dravida *et al*. showed that stem cells derived from human corneal-limbal stroma, expressed the ESC marker SSEA-4 (stage specific embryonic antigen-4) and other stem cell markers important for maintaining an undifferentiated state [45]. Therefore, LNS cells may become an ideal cell resource for single-factor reprogramming and subsequent retinal repair due to their existing stem/progenitor cell properties, multipotency and plasticity.

In summary, this data demonstrates the potential of mouse and human LNS to differentiate into retinal lineages *in* *vitro* and *in* *vivo*. The regulation of human LNS differentiation to a retinal lineage appears more comprehensive than with mouse LNS cells. As a readily accessible progenitor cell resource that can be derived from individuals up to 97 years of age, limbal neurosphere cells remain an attractive cell resource for the development of novel therapeutic approaches for degenerative retinal diseases.

## Supporting Information

File S1 Table S1, Primary antibodies used for immunocytochemical analysis. Table S2, Primer sequences used for phenotypic analysis and expected product sizes. Table S3, Primer and probe sequences for real time quantitative PCR analysis.
(PDF)

File S2 Mouse corneal limbus dissection.
(WMV)
