# Supplementary material for: Adult Limbal Neurosphere Cells: A Potential Autologous Cell Resource for Retinal Cell Generation
Source: PLoS One. 2014 Oct 1;9(10):e108418. doi: 10.1371/journal.pone.0108418 (PMC4182722; doi:10.1371/journal.pone.0108418)
Supplement: File S1 — Table S1, Primary antibodies used for immunocytochemical analysis. Table S2, Primer sequences used for phenotypic analysis and expected product sizes. Table S3, Primer and probe sequences for real time quantitative PCR analysis. (PDF) [file pone.0108418.s001.pdf]

## Supporting Information 1

**Table S1** Primary antibodies used for immunocytochemical analysis.

| **Antibody** | **Specificity** | **Company** | **Conc.** |
|---|---|---|---|
| Nestin | Neural stem cells | Chemicon | 1:100 |
| SOX2 | Embryonic germ cells/ neural stem cells | Chemicon | 1:100 |
| Pax6 | Ocular stem cells | Chemicon | 1:250 |
| class III β-tubulin (TuJ1) | Early differentiated neurons | Covance | 1:500 |
| Rhodopsin | Photoreceptors | Sigma Aldrich | 1:250 |
| Sytaxin3 | Synapse composition | Abcam | 1:500 |
| Recoverin | Photoreceptors | Abcam | 1:250 |
| GFAP | Glial cells | DAKO | 1:500 |

**Table S2** Primer sequences used for phenotypic analysis and expected product sizes.

| **Primers Name** | **Sequence** | **Size (bp)[a]** |
|---|---|---|
| Mouse_ K12_F | CTGTGGAGGCCTCTTTTCTG | **153** |
| Mouse_ K12_R | CCAGCTATCCCCATCCCTAT | |
| Mouse_ Lhx2 _F* | GCCATGCTGTTCCACAGTC | **509** |
| Mouse_ Lhx2 _R* | AAGTGCAAGCGGCAATAGAC | |
| Mouse_Pax6 _F* | CAGTTCTCAGAGCCCCGTAT | **489, 456** |
| Mouse_Pax6 _R* | CTAGCCAGGTTGCGAAGAAC | |
| Mouse_Rhodopsin _F | TCACCACCACCCTCTACACA | **216** |
| Mouse_Rhodopsin _R | TGATCCAGGTGAAGACCACA | |
| Mouse_Rhodopsin kinase_F | AGCCCGAGGAGAGAAGGTAG | **285** |
| Mouse_Rhodopsin kinase_R | CCCACGTCCTGAATGTTCTT | |
| Mouse_Tuj1_F | TGAGGCCTCCTCTCACAAGT | **207** |
| Mouse_Tuj1_R | CGCACGACATCTAGGACTGA | |
| Human_Lhx2_F | CAAGATCTCGGACCGCTACT | **284** |
| Human_Lhx2_R | CCGTGGTCAGCATCTTGTTA | |
| Human_Pax6_F | CGGAGTGAATCAGCTCGGTG | **300, 258** |
| Human_Pax6_R | CCGCTTATACTGGGCTATTTTGC | |
| Human_Rx_F | GAATCTCGAAATCTCAGCCC | **279** |
| Human_Rx_R | CTTCACTAATTTGCTCAGGAC | |
| Human_Rhodopsin_F | CACCACACAGAAGGCAGAGA | **378** |
| Human_Rhodopsin_R | AGGTGTAGGGGATGGGAGAC | |
| Human_GAPDH_F | ACCACAGTCCATGCCATCAC | **450** |
| Human_GAPDH_R | TCCACCACCCTGTTGCTGTA | |

[a] Base Pairs;

* Cycles for PCR: denaturing for 30 sec at 94°C; annealing for 30 sec at 60°C, extension for 30 sec at 72°C for 1 cycle; and denaturing for 30 sec at 94°C, annealing for 30 sec at 55°C, extension for 30 sec at 72°C for 34 cycles.

**Table S3** Primer and probe sequences for real time quantitative PCR analysis.

| **Primers Name** | **Sequence** | **Size (bp)** |
|---|---|---|
| Mouse_Rhodopsin _F | TCAGAAGGCAGAGAAGGAAGT | **109** |
| Mouse_Rhodopsin_R | CTGGTGGGTGAAGATGTAGAAG | |
| Probe | aTCTTCTTCCTGATCTGCTGGCTTCCCTACGaagaagat | |

Primers and probe were designed by PrimerDesign (PrimerDesign, Southampton, UK). Gapdh was used as reference gene (commercial primer/probe kit from PrimerDesign, sequence information are not available).

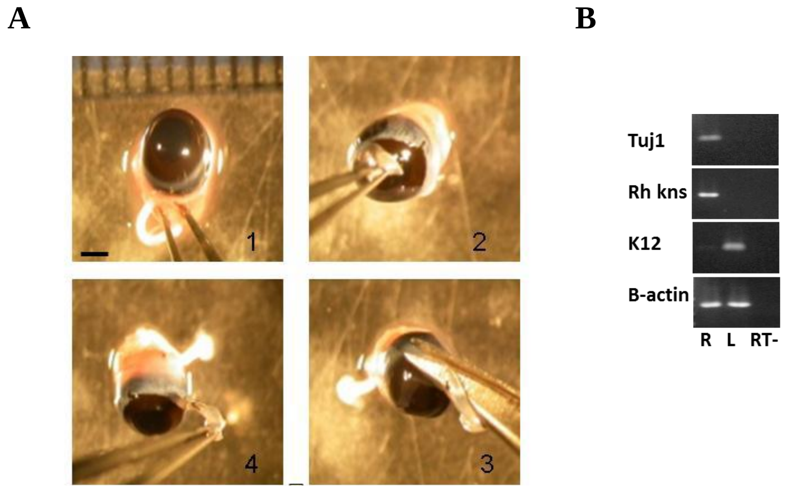


**Figure S1** Corneal limbus dissection overview. Adult mice (6-8 weeks) were used in this study. Following enucleation of the eyes and removal of the central cornea, a circular incision was made below the limbus to isolate the corneal limbal region, as shown in step 1-4 under microscopy (A). Cells were then dissociated by enzymatic means and cultured. Scale bar: 1.0 mm (B) No retinal markers were detected in the freshly isolated limbal cells. Tuj1: β-III tubulin, Rh kns: rhodopsin kinase, K12: K12 keratin, R: retina; L: freshly isolated limbal cells, Negative control (RT-) omitted reverse transcriptase.
